# Optimizing Perioperative Care for Ventricular Tachycardia Ablation in High-Risk Patients Supported by Impella 5.5

**DOI:** 10.7759/cureus.58642

**Published:** 2024-04-20

**Authors:** Nikhil Jaganathan, Mallikarjuna Devarapalli, Vikas Kumar

**Affiliations:** 1 Anesthesiology and Perioperative Medicine, Augusta University Medical College of Georgia, Augusta, USA

**Keywords:** impella monitoring, intracardiac echocardiography, perioperative, high-risk anesthesia, ventricular tachycardia ablation, cardiac anesthesiology, impella 5.5

## Abstract

Impella 5.5 (Abiomed Inc., Danvers, MA, USA) is a surgically implanted mechanical circulatory support device that helps support hemodynamically compromised patients. The device's risks and benefits must be entirely known, especially in the electrophysiology lab. Due to unexpected hemodynamic changes during pace mapping and ablation, such as ventricular tachycardia (VT) and asystole, it is sometimes necessary to implement chemical support with inotropic agents such as epinephrine or mechanical support with devices such as an Impella.

We present the case of a 72-year-old male with a biventricular implantable cardioverter-defibrillator (ICD) (Medtronic, Minneapolis, MN, USA) placed for refractory VT presenting for VT ablation. He had ischemic cardiomyopathy with a left ventricular ejection fraction (LVEF) of 33% and medical history of cardiac sarcoidosis, hypertension, hyperlipidemia, pulmonary embolism, left bundle branch block, and coronary artery disease. Due to the nature of the procedure and his history of arrhythmia, the patient was deemed a candidate for Impella 5.5. After evaluating patient risk factors, the cardiothoracic anesthesia team developed a strategic approach with imaging (including radiographic and echocardiographic imaging), Impella monitoring, and pharmacologic management with inotropes and vasopressors, allowing for uncomplicated perioperative management during the ablation. Given the procedure's intricacies and the patient's arrhythmia history, the medical team identified the patient as suitable for Impella 5.5 due to better performance and greater cardiac output than Impella 2.5 (Abiomed Inc., Danvers, MA, USA). Following a thorough assessment of the patient's risk factors, the cardiothoracic anesthesia team devised a comprehensive strategy to facilitate smooth perioperative management during the ablation, minimizing complications. The VT ablation procedure was performed successfully and effectively terminated the arrhythmia. However, the patient developed multifaceted postoperative complications, including cardiogenic shock, hemorrhagic shock, dyspnea, anemia, gastrointestinal abnormalities, and sepsis.

This case represents a highly complex patient scenario under the care of the cardiovascular anesthesiologist due to the nature of the procedure and numerous cardiovascular comorbidities, low ejection fraction, ICD placement, and malignant ventricular arrhythmia. We discuss the various perioperative management strategies and how they are tailored to such patients, including pharmacologic intervention, anesthesia administration, imaging modalities, and postoperative care. The purpose of this case report is to delineate the role of Impella 5.5 in perioperative care for high-risk VT ablation patients. We discuss the progression, pathophysiology, and management of this patient's multisystem complications following the procedure. We also highlight the use of Impella 5.5 in the electrophysiology lab and the anesthesia considerations, safeguards, and management strategies to optimize perioperative outcomes and avoid complications.

## Introduction

Ventricular tachycardia (VT) and ventricular fibrillation are the most common causes of sudden cardiac deaths, resulting in 300,000 annual deaths in the United States [1]. VT is a tachyarrhythmia involving a rate of 100 beats per minute for at least three sequential beats, which often arises from ischemic heart disease, dilated cardiomyopathy, infiltrative cardiomyopathy, electrolyte disturbances, cardiac channel defects, and structural heart defects [1]. In addition to antiarrhythmics, catheter ablation has high efficacy in treating VT. This procedure involves providing focused radiofrequency energy to damaged and fibrous areas of the heart, generating arrhythmic impulses for restoring normal sinus rhythm [1]. Maintaining hemodynamic stability for patients undergoing VT ablation is critical. VT ablation is a complex procedure that can lead to reduced cardiac function. During cardiac procedures, management of cardiogenic decompensation often includes treatment based on underlying causes or providing pharmacological interventions, such as inotropes and vasopressors [2]. Alternatively, an Impella (Abiomed Inc., Danvers, MA, USA) is a transvalvular heart pump that provides continual flow by aspirating blood from the left ventricle and propelling blood into the aorta, providing temporary circulatory support during cardiac compromise [2]. Complications of Impella include aortic insufficiency and reduced flow rate due to insufficient preload [2,3]. Improper Impella placement can lead to arrhythmia, hypoperfusion, and bleeding [2,3]. In contrast to Impella 2.5 (Abiomed Inc., Danvers, MA, USA) and Impella Cardiac Power (CP) (Abiomed Inc., Danvers, MA, USA), which are implanted percutaneously through the femoral artery, Impella 5.5 is placed surgically and is capable of greater maximum flow of up to 6 L/min [2]. We detail the flow rate, position sensing, and other differences between the devices in the "Discussion" section. 

Anecdotally, anesthetic management of VT ablation procedures is highly complex due to the risk of asystole and cerebral hypoperfusion, especially in a setting of systolic dysfunction. While prior iterations of Impella devices have been studied extensively, surgically implanted Impella 5.5 is increasingly used in the electrophysiology lab, necessitating examination of perioperative and post-procedural outcomes. In this case, we present a patient undergoing VT ablation with general anesthesia in the electrophysiology lab. The existing literature regarding Impella 5.5 (Abiomed Inc., Danvers, MA, USA) and considering anesthesia for high-risk Impella patients during electrophysiology procedures is currently limited. Moreover, the current absence of standardized guidelines regarding monitoring and weaning patients from Impella posits the need for a comprehensive analysis of perioperative factors [2]. Greater evaluation of Impella 5.5 efficacy in VT ablation compared to prior generations of Impella, especially among patients based on comorbidities, is crucial to gauge postoperative recovery time and complications. Furthermore, based on our literature review, no case reports have been published that highlight the perioperative anesthesia considerations and management of high-risk Impella 5.5 patients undergoing VT ablation. 

In this case, Impella 5.5 was elected for the patient to increase the chances of successful ablation and provide better hemodynamic stability [4]. We discuss the anesthetic implications, concerns, and procedural strategies for a high-risk patient with numerous cardiovascular comorbidities and biventricular implantable cardioverter-defibrillator (ICD) placement. Furthermore, we highlight the role of the recently introduced Impella 5.5 in maintaining hemodynamic stability in VT ablation. Ultimately, the case report aims (1) to illustrate the use of Impella 5.5 in the perioperative setting of the electrophysiology lab for high-risk VT ablation patients, (2) to delineate multifactorial sequelae following Impella 5.5 placement, and (3) to convey the anesthesia considerations and strategies to facilitate optimal perioperative outcomes. 

## Case presentation

In summary, the patient is a 72-year-old male with a complex medical history with cardiovascular comorbidities and low ejection fraction presenting with VT. Diagnostic imaging revealed dilated cardiomyopathy and cardiac sarcoidosis. As a result of persistent VT refractory to amiodarone, he underwent Impella 5.5 placement for VT ablation. General anesthesia, hemodynamic support, and Impella monitoring were provided intraoperatively. The patient experienced cardiopulmonary compromise, hemorrhagic shock, dyspnea, anemia, gastrointestinal abnormalities, and sepsis postoperatively. Despite challenges, with aggressive management, the patient was discharged without long-term complications. 

The patient is a 72-year-old white male with a past medical history significant for hypertension, hyperlipidemia, chronic obstructive pulmonary disease, deep vein thrombosis, pulmonary embolism, coronary artery disease, percutaneous coronary intervention with a drug-eluting stent, ischemic cardiomyopathy, and left bundle branch block. Furthermore, this patient previously had a measured left ventricular ejection fraction (LVEF) of 33%. The patient reported a significant smoking and alcohol history and use of continuous positive air pressure (CPAP) at home. 

The patient presented with VT cardiac arrest, after which he received implantation of a Medtronic dual-chamber ICD. Two months later, the patient had a subsequent episode of VT and presented with non-ST-elevation myocardial infarction (NSTEMI). The patient was discharged but later returned to the emergency department, where he presented with several episodes of VT treated with amiodarone. A chest computed tomography (CT) scan without contrast showed several calcified pulmonary parenchymal granulomas and calcified mediastinal lymph nodes, indicating prior granulomatous disease. Furthermore, cardiac magnetic resonance imaging (MRI) revealed findings of cardiac sarcoidosis. 

Our patient presented to the emergency department with chest tightness and palpitations. Device interrogation of his ICD demonstrated VT, requiring multiple shocks from the device. The patient's hospital course was complicated by four episodes of VT requiring amiodarone, resulting in the patient being admitted to cardiology with electrophysiology consultation for rhythm management, upon which a decision for VT ablation was made. However, since the VT was persistent and refractory to amiodarone treatment, the patient was selected as a candidate for VT ablation. 

Transthoracic echocardiography (TTE) performed three weeks prior to the procedure found mild concentric left ventricular hypertrophy with global hypokinesis and diastolic dysfunction. Furthermore, TTE results demonstrated mildly dilated right ventricular size, mildly dilated left atrium, moderately dilated right atrium, and mild mitral and tricuspid regurgitation. Right ventricular systolic pressure was calculated at 41 mmHg with mild pulmonary hypertension. The inferior vena cava was found to be dilated, and aortic and mitral valves were calcified. Additionally, positron emission tomography (PET) imaging one week pre-procedure demonstrated calcified granulomatous nodes in the lower paraesophageal region and bibasilar atelectasis, notable in the left lung with bibasilar pleural thickening, further supporting the diagnosis of sarcoidosis. Due to low ejection fraction, dilated cardiomyopathy, and systolic dysfunction identified by TTE, the patient was selected for Impella placement to facilitate adequate intraoperative perfusion. 

This patient had no history or family history of complications from anesthesia. The anesthetic plan, risks, benefits, alternatives, and complications were discussed with the patient. Vital signs included a temperature of 37°C, a heart rate of 62 beats per minute, a blood pressure of 109/50 via the arterial line, and a SpO2 of 99%. 

One day prior to the VT ablation procedure, Impella 5.5 was implanted by the cardiothoracic surgeon. Fluoroscopy and transesophageal echocardiogram were utilized for the implantation of the Impella 5.5 device. The said device was then inserted over the guidewire into the left ventricle. The Impella device position was withdrawn slowly into the aortic root while securing an adequate length (5 centimeters) of the device below the aortic valve, confirmed using transesophageal echocardiography for the satisfactory position of the inflow into the device and outflow from the device sufficient distance above the aortic valve. The Impella device support was initiated and progressively escalated to provide systemic outflow >3.0 L/min with the P6 setting. The device function was satisfactory. The device developed a suction alarm due to relative hypovolemia, and the speed was adjusted to P4, maintaining a flow of 2.2-2.3 L/min. There were no pericardial effusion and no other concerning findings on the echocardiogram. 

The periprocedural TEE demonstrated LVEF of 30% with mildly reduced right ventricle function, moderate mitral valve insufficiency, and moderate tricuspid valve insufficiency. The TEE showed the Impella device directed towards the apex and 5 centimeters deep into the left ventricle from the aortic valve. The Impella function was satisfactory on P4 with appropriate waveforms, CPO, and no suction alarms. 

Regarding anesthesia-specific procedures during the ablation process, arterial line placement allowed for continuous and accurate hemodynamic monitoring, while femoral central venous access facilitated rapid fluid administration. Because this patient has potentially high hemodynamic fluctuation, an arterial line was administered to monitor blood pressure. Since this patient is at increased risk of hemorrhage due to the surgically implanted Impella 5.5, arterial line placement is especially crucial. Additionally, central venous access was established to administer vasopressors and monitor central venous pressure. 

**Figure 1:**
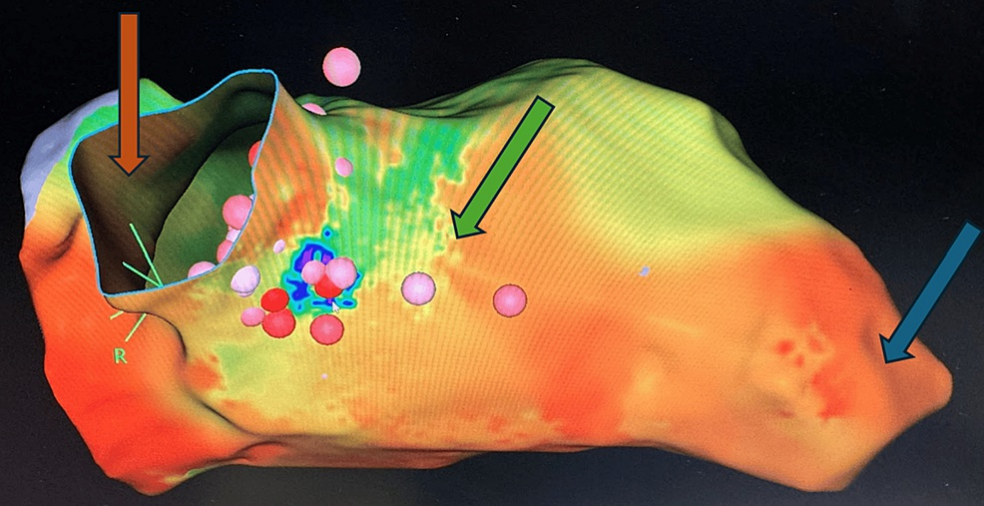
Three-dimensional electroanatomic mapping of the patient's heart was utilized to facilitate ventricular tachycardia localization and ablation. Notable anatomic features include the aorta (orange arrow), interventricular septum (green arrow), and apex ventricle (blue arrow). Electroanatomic mapping found the ventricular tachycardia to originate from the basal aspect of the heart anteriorly, allowing for localized ablation.

The patient was induced and intubated with propofol, fentanyl, etomidate, and rocuronium. The rationale for induction with etomidate as the primary hypnotic agent and low dose of propofol was to prevent hemodynamic collapse due to the vasodilatory effect of propofol alone and to avoid the delayed induction of etomidate; however, the primary induction agent used was etomidate due to the patient's significantly low ejection fraction. During the procedure, the patient was maintained on volatile agents and remifentanil for pain management. The patient also received bicarbonate and calcium chloride boluses to compensate for probable metabolic acidosis due to reduced perfusion. The patient received multiple boluses and an infusion of epinephrine to maintain hemodynamic stability. As shown in Figure 1, the electrophysiologist utilized pace mapping and 3D electroanatomic mapping to locate the focus of VT, which underwent radiofrequency ablation. At the beginning of the procedure, the patient was in sinus rhythm. During ablation, the electrophysiologist detected one monomorphic VT at the cycle length of 450 milliseconds with right bundle branch morphology, consistent with the clinical tachycardia. The VT originated from the basal aspect of the heart anteriorly. Mapping in VT in that area resulted in about 70% of the tachycardia cycle length noted to be within a 5-mm distance, and the ablation catheter was inserted into the left ventricle to ablate the arrhythmia source. The duration of the procedure was approximately two hours. At the end of the procedure, protamine was administered for heparin reversal, sugammadex for neuromuscular blockade reversal, and ondansetron for nausea prophylaxis.

Regarding the anesthesia considerations taken to ensure an uncomplicated procedure, the Impella console was closely monitored to gauge various factors, including aortic pressure, motor speed, cardiac output, left ventricular function, and a purge system to modify the volume and pharmacologic infusion. The use of tailored strategies allowed for reasonable flow, motor speed, and aortic pressure with acceptable fluctuations. Impella 5.5 was set to a performance level (P-level) of 4 to maintain continuous perfusion. Adequate perfusion was assessed by gauging lactate, oxygen saturation, blood pressure, cardiac output, and urine output. 

**Figure 2:**
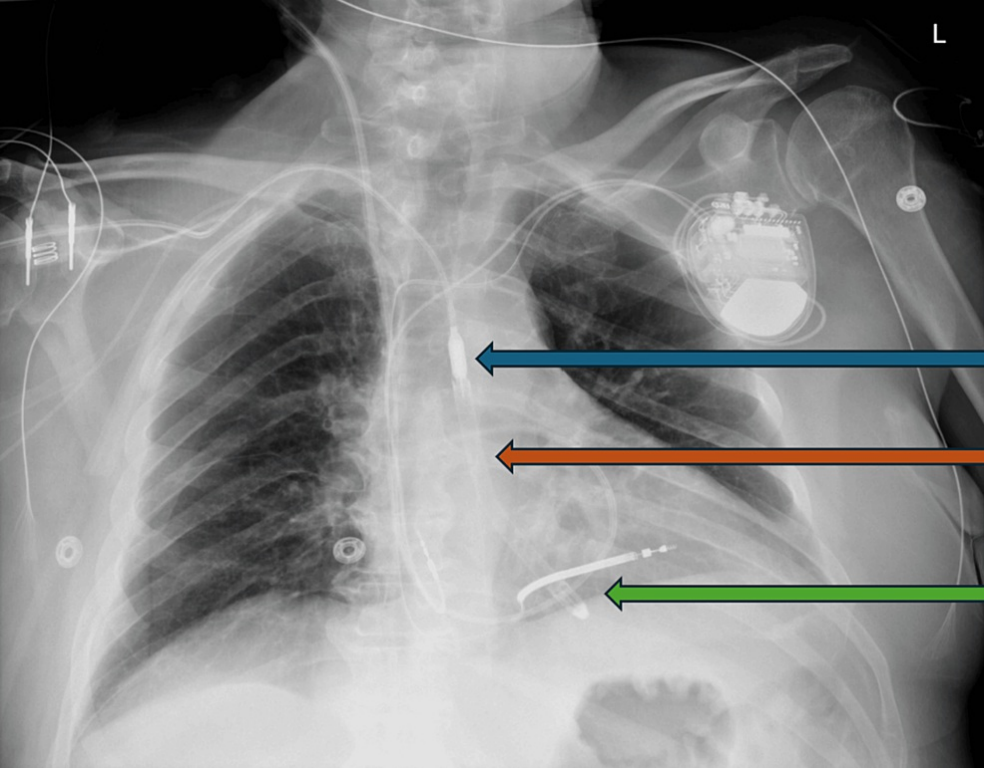
Chest X-ray was obtained to confirm appropriate Impella placement across the aortic valve and into the left ventricle. The Impella outflow (blue arrow), approximate position of the aortic valve (orange arrow), and left ventricle (green arrow) can be observed. The Impella device is directed towards the apex and 5 centimeters deep into the left ventricle from the aortic valve.

No anesthetic complications were noted after the ablation, and the patient was stable. After transfer to the post-anesthesia care unit, the patient initially became hypotensive, was placed on pressors, and became anemic and dyspneic, requiring a high-flow nasal cannula. The post-procedure course was complicated by hemorrhagic shock due to unstable active extravasation from a branch artery arising from the right profunda femoris artery, requiring multiple units of red blood cells and intubation, and interventional radiology was consulted for embolization of the branch artery. The patient was weaned off vasopressors and maintained on Impella at a P-level of 4. As shown in Figure 2, a chest X-ray and transthoracic echocardiogram were performed to evaluate the position of the Impella, right ventricular function, and signs of tamponade. Chest X-ray confirmed the placement of the right internal jugular approach pulmonary artery catheter with its tip overlying the right pulmonary artery and the right subclavian approach Impella device overlying the central left ventricle. 

Impella removal was performed on postoperative day 3, which the patient tolerated well. Prior to Impella removal, the patient demonstrated a cardiac index (CI) of 3.0 L/min/m^2^ and cardiac output of 6.54 L/min. Following Impella removal, the patient demonstrated a CI of 2.96 L/min/m^2^, cardiac output of 6.46 L/min, and central venous pressure (CVP) of 9 mmHg. Thus, following Impella removal, the patient was initially in a hemodynamically stable state. The patient was provided two units of packed red blood cells for low hemoglobin of 6.6 g/dl. Additionally, he was treated with diuretics to eliminate fluid overload secondary to intravenous fluid and multiple blood transfusions he received during his hospital course. 

After transfer to the cardiovascular intensive care unit (CVICU), the patient developed progressively worsening cardiogenic shock on vasopressors with an ejection fraction of 30%, gradually becoming hypotensive, anemic, thrombocytopenic, and dyspneic. The patient's lactate levels increased from 1.5 to 2.8 mmol/L, while hemoglobin dropped from 11 to 8.5 g/dl. 

The patient's blood pressure declined to 80/40 mmHg, pulmonary artery pressure increased from approximately 20/10 mmHg to 40/20 mmHg, and venous oxygen saturations trended down from 50s to a low of 37. Dobutamine was started to promote cardiac contractility and increase cardiac output. Norepinephrine, vasopressin, and epinephrine were infused to maintain blood pressure with heparin for anticoagulation. The critical care providers attempted to increase Impella flow from P4 to P6, but the patient immediately had a suction event and did not tolerate the increase. A suction event involves reduced pump outflow due to obstruction of the inlet, which can be due to improper device positioning, hypovolemia, thrombus, or right ventricular failure, and is often resolved by lowering the flow level [2]. 

Echocardiography demonstrated severely reduced left ventricular function, mildly reduced right ventricular function, dilated right ventricle, the collapse of the inferior vena cava with respiration, and some anterior pericardial effusion. Left infrahilar/basilar airspace opacity on chest X-ray was interpreted as atelectasis. Additionally, the patient reported dyspnea on a 100% nasal cannula and requested intubation. The patient was intubated for one day with continued pressor support. The pulmonary artery catheter revealed low pulmonary artery pressures and low central venous pressure. Although the Impella device functioned well on P4 at 2.1-2.2 L/min, increasing speed to P5 quickly demonstrated a suction alarm due to hypovolemia, so the patient was returned to P4 flow. 

In addition to cardiopulmonary compromise, the patient developed multiple other complications in the CVICU. Chest CT angiography demonstrated a small eccentric filling of the proximal innominate artery, which was suspected to be an eccentric thrombus. Pelvis CT angiography demonstrated expanding intramuscular hematoma within the right adductor and intramuscular hematoma in the right iliopsoas. Additionally, the patient developed constipation and diarrhea within one day following the ablation procedure, and increased mucosal enhancement of the proximal duodenum on pelvic angiography indicated inflammatory changes compatible with duodenitis. The patient developed short-term metabolic acidosis with bicarbonate levels reduced from 23 mmol/L to 19 mmol/L, which gradually resolved. The patient's CVICU course was also complicated by suspected sepsis with fever, hypotension, and leukocytosis, necessitating vancomycin and cefepime, yet blood cultures and bronchoalveolar lavage yielded negative results. Although the patient had persistent pulmonary edema and cardiomegaly, he developed gradual improvement of airspace opacifications. 

The patient was discharged home from the hospital six days after Impella removal and experienced no long-term complications from the procedure. The patient has been stable at one-month and two-month cardiology follow-up appointments with no post-procedural complications. 

## Discussion

Perioperative management strategies

Care for patients on Impella support necessitates various perioperative considerations. The use of echocardiography can be highly beneficial to determining right ventricular function, monitoring for device-associated complications, and confirming appropriate device positioning [5]. As used in this patient, transesophageal echocardiography with fluoroscopy is typically utilized to facilitate implantation. The TTE is utilized to ensure proper placement of the guidewire in the aorta lumen across the aortic valve and reaching the left ventricle with its tip facing the apex, typically visualized via mid-esophageal long-axis view [2]. While the Impella 5.5 must be inserted 5 cm into the left ventricle from the aortic valve, other Impella devices proceed 3.5 cm into the ventricle [2]. Additionally, TTE should be utilized to ensure central placement in the ventricle and the absence of injury to the mitral valve, aortic valve, and aorta, which can minimize arrhythmia and suction events [2]. Once the Impella device is positioned adequately, the pump flow slowly begins to allow the right ventricle to adjust to the increased preload, and Color Doppler is utilized to ensure flow from the left ventricle into the aorta through the inlet and outlet [2].

In addition to intracardiac echocardiography, pulmonary artery catheter and arterial blood pressure monitoring can be used to ensure optimal management of patients on Impella [5]. Retrospective registry data analyses have found pulmonary artery catheter monitoring for hemodynamic evaluation before and during transvalvular heart pump support correlated with improved survival, making it a critical part of the perioperative evaluation [2]. To prevent inadequate preload and ventricular dysfunction, a number of factors need to be taken into account. These include monitoring for alterations in ventricular size, pulmonary capillary wedge pressure (PCWP), ventricular dilatation, ventricular contractility, interventricular septum shifts, and mitral regurgitation [5]. During placement of the device, heparin is typically infused to establish a clotting time of 250 seconds to reduce the risk of intraoperative thrombosis [2]. 

Our patient presented with multiple cardiovascular comorbidities and a significantly low ejection fraction, increasing the risk of anesthesia. Furthermore, the presence of ventricular arrhythmia, potentially arising from sarcoidosis or coronary artery disease, posed a unique challenge. Patients with a low ejection fraction are unlikely to tolerate ablation, necessitating Impella placement to allow for circulatory support and, thus, the maintenance of cerebral perfusion. Stimulating the heart at a low ejection fraction can be poorly tolerated, leading to asystole, and thus, left ventricular assist device implantation can at least facilitate cerebral perfusion even when systemic perfusion is compromised. Due to ischemic cardiomyopathy and malignant arrhythmia, the electrophysiology team and cardiothoracic surgeon elected to insert an Impella 5.5 device, as the patient was vulnerable to hemodynamic instability. As the patient was refractory to medications and ICD therapy, ventricular catheter ablation was chosen. 

Many tendencies in postoperative sequelae are common and can be predicted to optimize patient outcomes. Fluid overload due to intraoperative blood transfusions is common, often necessitating diuretics, especially since post-procedure red blood cell transfusions are needed due to blood loss. If PCWP and CVP are increased with normal CI, the patient may have excess fluid, while low PCWP and CVP may indicate low intravascular volume [2]. Additionally, cardiogenic shock is a common and life-threatening complication of electrophysiology ablation procedures, which can be managed with dobutamine, norepinephrine, vasopressin, and epinephrine for increased inotropy and vascular tone as well as administration of fluids. Cardiogenic shock and left ventricular failure can be indicated by cardiac power (index of cardiac output) lower than 0.6 W, increased pulmonary capillary wedge pressure, ventricular dilation, increased mitral regurgitation, and reduced ventricular contractility on echocardiography [2]. Multiple attempts to increase the P-level of the Impella from P4 to P5 or P6 to promote greater perfusion were not tolerated. They resulted in a suction event, suggesting that a P-level of P4 may be optimal in similar patients, and caution should be exercised when manipulating the P-level in the postoperative setting. In general, a pulsatility that brings CI, central venous pressure, and pulmonary capillary wedge pressure with normal ranges is utilized, and significantly increased PCWP can be an indication of greater ventricular support [2]. 

Transitioning a patient off of an Impella device is typically initiated when mean arterial pressure is 65 mmHg and heart rate is lower than 100 beats per minute with less than moderate levels of device pulsatility and pharmacologic agents, indicating stable hemodynamics with improved perfusion (lactate concentration less than 2 mmol/liter) [2]. In our case, the Impella device was removed by progressively decreasing the pulsatility flow of the Impella with epinephrine and vasopressors to aid perfusion and allow for device removal. Impella removal was monitored without assistance by echocardiography and a pulmonary artery catheter to assess cardiac function. The patient demonstrated a CI of 3.0 L/min/m^2^ and cardiac output of 6.54 L/min before Impella removal. Subsequently, the patient demonstrated a CI of 2.96 L/min/m^2^, cardiac output of 6.46 L/min, and central venous pressure of 9 mmHg after Impella removal. The relatively stable CI and cardiac output indicate that the patient successfully transitioned from the Impella device and was initially in a hemodynamically stable state. 

Additionally, frequent imaging, including echocardiography and X-ray, are critical to gauge systolic function and dilation, pericardial effusion, and atelectasis. Pericardial effusion may also be detected on the Impella console as increased central venous pressure without equalization of pressures [2]. Dyspnea following Impella-based procedures can be multifactorial, including cardiogenic shock exacerbation, pericardial effusion, and atelectasis, and can be managed by intubation if refractory to supplemental oxygen. Since transvalvular heart pumps can increase oxygenation by promoting left ventricular emptying and decreasing pulmonary edema, Impella removal may precipitate dyspnea [2]. Moreover, hemorrhagic shock due to bleeding from the right profunda femoris artery and intramuscular hematoma of the right iliopsoas necessitate embolization and blood transfusion. Hypovolemic shock can lead to hypoperfusion and reduced preload, which can be detected by low-end-diastolic volumes, reduced ventricular size, and decreased filling pressures [2].

Additionally, renal function must be frequently evaluated since cardiogenic and hypovolemic shock may result in diminished perfusion and metabolic acidosis, such as in our patients. In the setting of any cardiac assist device implantation, sepsis and gastrointestinal complications must be monitored as well. Ultimately, this array of short-term sequelae was aggressively and effectively managed, allowing for prompt discharge and no long-term complications. 

Literature review

The efficacy of Impella use during VT ablation is yet to be described. Miller et al.'s prospective study evaluated the results and hemodynamic effects of patients undergoing VT ablation who were given hemodynamic support by Impella 2.5, a type of percutaneous ventricular assist device (pLVAD) [6]. After studying 20 patients with scar-related VT ablation, the researchers concluded that pLVAD was a safe way to facilitate scar VT ablation in patients with severe left ventricular dysfunction. The study used cerebral oximetry to measure the levels of cerebral desaturation. It showed that cerebral desaturation to less than or equal to 55% occurred during ventricular ablation in 53% of patients without pLVAD support compared to 5% of patients with pLVAD support [6]. However, multiple studies have found no significant difference in postoperative outcomes and survival in patients with Impella support for patients undergoing ablation of VT, with a high incidence of complications attributed to the device [7,8]. 

On the other hand, the Impella 5.0 and 5.5 placement necessitates a more invasive approach, frequently involving direct surgical insertion into the ascending aorta [9]. Previous generations of the Impella devices, namely, the Impella 2.5 and Impella CP, are placed percutaneously through the femoral artery into the ascending aorta and then to the left ventricle. The Impella 5.5 offers numerous advantages compared to previous iterations of these devices, such as greater flow rate, more precise position monitoring, enhanced hemocompatibility, reduced motor size, and a lack of a pigtail catheter [10,11]. In contrast, the pigtail catheter of Impella 2.5, CP, and 5.0 is a distinguishing feature from Impella 5.5 and is used to provide stability during implantation [2]. In comparison to the fiber-optic sensor of Impella 5.5, a fluid-filled pressure lumen in the CP and 2.5 and a differential pressure sensor in the 5.0 are used for device positioning [2]. Additionally, the flow rate of Impella 5.5 (6 L/min) is greater than that of Impella 2.5 (2.5 L/min), Impella CP (4.3 L/min), and Impella 5.0 (5.0 L/min) [2]. Although preliminary data indicates positive outcomes for Impella 5.5, current published data comparing Impella 5.5 to previous generations is very limited [10]. 

The novel retrospective study by Sroubek et al. involved patients at risk of perioperative circulatory complications requiring ventricular fibrillation who received Impella 5.0 and 5.5 [8]. The sample included 16 patients receiving Impella 5.0 and 25 receiving Impella 5.5. After 423 days following the ablation procedure, 59% of the control patients and 54% of the Impella patients died or required a heart transplant or permanent left ventricular assist device, with a comparable recurrence of arrhythmia between the groups [8]. Ultimately, the researchers concluded that Impella 5.0 and 5.5 allowed for VT ablation procedures but did not play a significant role in post-procedural survival, ventricular fibrillation recurrence, death, the need for pLVAD implantation, or heart transplantation [8]. This study prompts more investigation into the indications of Impella 5.5 compared to prior generations and whether individualized patient factors may determine the optimal device. 

Our literature review yielded two case reports of Impella 5.5 usage in the context of VT ablation. A patient who underwent coronary bypass grafting complicated by sustained VT ablation is presented in the Volgmann et al. case report [12] as having been admitted for NSTEMI. Volgmann et al. highlight that surgical axillary placement of Impella 5.5 is necessary to maintain hemodynamic stability in pulmonary congestion and left ventricular distension [12]. In this case, due to persistent ventricular arrhythmias following the ablation and decreased right ventricular function, the patient was deemed a candidate for heart transplant and was stabilized on Impella 5.5 as a bridge-to-transplant approach. Additionally, the case report by Bhardwaj et al. describes the case of a patient with an LVEF of 35% presenting with persistent VT with symptoms of intravascular hemolysis and acute kidney injury, resulting in an upgrade from Impella CP to Impella 5.5, thereby providing adequate hemodynamic stability and left ventricular outflow [13]. Impella 5.5, in this case, allowed for rescue from end-organ dysfunction and successful VT ablation [13]. The cases delineated by Volgmann et al. and Bhardwaj et al. showcase the versatility of Impella 5.5 in cardiac dysfunction and its applications to the electrophysiology lab [12,13]. These cases demonstrate the usage of Impella 5.5 in arrhythmia patients in which prior Impella generations may be inadequate [12,13]. Anecdotally, we have found similar results with poorer outcomes with percutaneous devices such as Impella CP compared to Impella 5.5 for VT ablation. These two case reports share our patient's refractory VT and cardiovascular comorbidities, including significantly low ejection fraction, and highlight the value of Impella 5.5 in promoting intra-procedural perfusion during ablation, suggesting better outcomes and the need for increased use of Impella 5.5 in such complex procedures. 

An additional case report by Patel et al. describes the case of a 60-year-old male with dilated cardiomyopathy, a candidate for heart transplant, who presented with a VT storm that persisted despite amiodarone and lidocaine administration with resultant cardiogenic shock [14]. In contrast to the two prior case reports described, this patient was deemed high risk for VT ablation but received implantation of Impella 5.5 for 34 days as a bridge to heart transplant [14]. Therefore, Impella 5.5 is indicated for hemodynamic support for VT ablation and transitional support before heart transplantation [12-14]. Thus, the case report by Patel et al. differs from our patient as this case report describes a patient with cardiogenic shock directly resulting from VT, hence necessitating Impella 5.5 for a different purpose, in contrast to the intraoperative device during the ablation procedure as in our patient. Therefore, due to refractory VT, Impella 5.5 may play a dual role in facilitating VT ablation procedures and heart transplants. 

Implications for clinical practice

The Impella 5.5 is not yet a standard for electrophysiology procedures, and its implantation requires a more invasive surgical technique than the previous generations' percutaneous placements [3]. However, the high-risk nature of this procedure warranted a higher maximum flow rate to ensure optimal circulatory function. Thus, Impella 5.5 can be a valuable alternative for pLVADs, extracorporeal membrane oxygenation (ECMO), and intra-aortic balloon pumps. Intracardiac echocardiography and Impella monitoring, coupled with anticoagulants, vasopressors, and fluids as needed, allowed for successful ablation without anesthetic complications. As Impella 5.5 becomes a more central component of perioperative care in the electrophysiology lab in the foreseeable future, it is fundamental that anesthesia providers are equipped with the considerations and strategies to facilitate an uncomplicated procedure in such high-risk patients. 

The findings of this case are highly generalizable to broader patient populations with lessons that can be extrapolated not only to other patients undergoing VT ablation with Impella 5.5, but some insights can be applied to similar electrophysiology procedures or prior Impella devices. Although our patient presented with unique cardiovascular comorbidities and postoperative sequelae, which make this case especially challenging and limit generalizability to an extent, a thorough evaluation of this case furthers our understanding of optimal perioperative management for high-risk patients undergoing VT ablation. 

Standardized intraoperative monitoring protocols for transvalvular heart pump therapy patients have not been established [2]. Reflection of this case reveals various clinical applications, including the diagnostic workup and identification of refractory VT and candidacy for Impella 5.5. This case also highlights the importance of analyzing Impella console factors (aortic pressure, motor speed, CI, cardiac output, left ventricular function, and purge system) to alter volume and pharmacologic infusion, the role of adjusting the P-level to maintain hemodynamic stability, and the need for monitoring CI and cardiac output as a measure of cardiac function post-Impella. Moreover, this report highlights the use and rationale of various perioperative management strategies, including arterial line placement, femoral central venous access, and administration of medications for induction and maintenance of anesthesia. Additionally, we highlight the critical role of imaging, including TTE and chest X-ray, for evaluating pre-procedural and post-procedural cardiac and pulmonary function, including ejection fraction, cardiomegaly, valve calcification and regurgitation, vena cava dilation, and pulmonary opacities and atelectasis. Lastly, our extensive discussion of the multifactorial post-procedural sequelae has high clinical relevance in the critical care setting. It can be used to predict short-term complications in similar patients. Notably, a comprehensive, multidisciplinary team involving cardiac anesthesiologists, electrophysiologists, and cardiothoracic surgeons during the procedure, followed by care from critical care anesthesiologists and ICU specialists afterward, is fundamental. 

## Conclusions

This patient represents a medically challenging patient under the care of a cardiothoracic and critical care anesthesiologist and underscores the comprehensive analysis of factors essential for the stability of a hemodynamically vulnerable patient. Although Impella use presents complexities to the anesthesiologist, thorough perioperative considerations and versatile anesthesia strategies allow for the effective management of patient hemodynamic outcomes. Further randomized clinical studies on VT ablation using Impella 5.5 for hemodynamic support will be needed to provide more insight into post-procedural patient outcomes and to provide definitive indications for Impella 5.5. Since our case report presents a patient with extensive short-term complications and limited long-term complications, avenues for further investigation may incorporate a temporal aspect into Impella results to gauge the span of complications post-procedure. An area of uncertainty in our patient was the reasons for the patient's hemodynamic instability post-procedure, especially considering the initial stability with Impella support and progressive deterioration despite aggressive intervention. Future investigation can analyze the occurrence of cardiogenic shock following VT ablation across various comorbidities, cardiac function, and Impella devices to minimize this noteworthy complication. 

In essence, a thorough diagnostic workup, analysis of Impella and cardiac function factors, precise anesthesia and pharmacologic interventions, and frequent imaging are fundamental for the care of such challenging patients. In such a case, a multidisciplinary approach is necessary to manage complex arrhythmias, with collaboration among cardiac anesthesiologists, electrophysiologists, and cardiothoracic surgeons during the procedure followed by careful management of critical care anesthesiologists and ICU specialists in the post-procedural period. As Impella 5.5 continues to be studied and potentially a mainstay in the electrophysiology lab, careful consideration of numerous anesthesia variables and post-procedural sequelae must be evaluated to ensure effective perioperative management of VT ablation patients and, thus, optimal outcomes. 
